# Nitric oxide regulates the expression of heme carrier protein-1 via hypoxia inducible factor-1α stabilization

**DOI:** 10.1371/journal.pone.0222074

**Published:** 2019-09-12

**Authors:** Hiromi Kurokawa, Hiromu Ito, Masahiko Terasaki, Daisuke Matano, Atsushi Taninaka, Hidemi Shigekawa, Hirofumi Matsui

**Affiliations:** 1 Faculty of Medicine, University of Tsukuba, Ibaraki, Japan; 2 Graduate School of Medical and Dental Sciences, Kagoshima University, Kagoshima, Japan; 3 Graduate School of Comprehensive Human Sciences, University of Tsukuba, Ibaraki, Japan; 4 Faculty of Pure and Applied Sciences, University of Tsukuba, Ibaraki, Japan; University of Nebraska Medical Center, UNITED STATES

## Abstract

Photodynamic therapy (PDT) is a cancer therapy that capitalizes on cancer-specific porphyrin accumulation. We have investigated this phenomenon to propose the following three conclusions: 1) the mechanism underlying this phenomenon is closely related to both nitric oxide (NO) and heme carrier protein-1 (HCP-1), 2) NO inactivates ferrochelatase, and thus, the intracellular porphyrin levels in the cells are increased by the administration of an NO donor after 5-aminolevulinic acid treatment, 3) HCP-1 transports not only heme but also other porphyrins. Since NO stabilizes hypoxia-inducible factor (HIF)-1α, resulting in the upregulation of heme biosynthesis, HCP-1 expression can be increased by HIF-1α stabilization. In this study, we determined whether NO regulates HCP-1 expression by stabilizing HIF-1α expression. For this purpose, rat gastric cancer cell line RGK36 was treated with L-arginine or N6-(1-iminoethyl)-L-lysine (L-NIL). L-arginine treatment increased the intracellular NO concentration, and both HCP-1 and HIF-1α expression, while L-NIL treatment decreased them. Cytotoxicity of PDT was enhanced by L-arginine, following intracellular hemato-porphyrin dihydrochloride (HpD) accumulation. Both Cytotoxicity of PDT and HpD accumulation were decreased by L-NIL. The HCP-1 and HIF-1α expression, intracellular HpD accumulation and PDT cytotoxicity were decreased by 2-methoxyestradiol, which is a HIF-1α inhibitor. Moreover, these phenomena were not increased by a combination of both L-arginine and 2-Me. Thus, HCP-1 can be a downstream target of HIF-1α. These effects were also induced in the human gastric cancer cell line MKN45. Taken together, we conclude that HCP-1 expression is regulated by NO via HIF-1α stabilization.

## Introduction

Porphyrins are substrates used for photodynamic therapy (PDT), a cancer treatment using the combination of a photosensitizer and laser irradiation [1]. The advantages of this therapy include reduced treatment time, minimal normal tissue toxicity, no long‐term systemic toxicity and lack of drug resistance mechanisms [2, 3]. PDT is used the capability of combination with chemotherapy make it more commonly used in many fields of medicine [3]. Developed countries are evolving into super-aged socieites, i.e. socieites with a larger porportion of population in the 65 and older age group such as Japan, German, and Italy [4]. PDT is desired as a less invasive treatment because it has no requirement for anesthesia and a lower risk of bleeding than with surgery [2]. In these conditions, the usefulness of PDT has been re-recognized [5]. The fundamentals of this therapy involve the cancer-specific accumulation of porphyrins [6]. Lee et al. reported that porphyrin accumulation and PDT effect were different between normal and cancer skin sell line [7] Jedrych et al. reported that cell viability after PDT treatment in normal mouse embryo was higher than that in human lung carcinoma [8]. However, the mechanism of this accumulation remains unknown, and many clinicians have disputed the ability of cancer to accumulate porphyrins specifically. Thus, PDT has not become a major form of cancer therapy.

Heme carrier protein-1 (HCP-1) is a proton-coupled folate transporter (SLC46A1) that transports heme into cells [9, 10]. We have reported that cancer-specific accumulation of porphyrins is involved in this heme transport because the structure of porphyrin is virtually the same as that of heme [11, 12]. We confirmed that HCP-1 expression is cancer-specific and that hematoporphyrin fluorescence is higher in HCP-1-overexpressing HeLa cells than in wild-type cells after treatment with hematoporphyrin [11]. Cell viability after PDT is significantly lower in HCP-1-overexpressing HeLa cells compared to control cells. In contrast, when HCP-1 was silenced with siRNA, the intracellular fluorescence intensity of hematoporphyrin was decreased. From these results, we concluded that HCP-1 is a cancer-specific porphyrin transporter. Moreover, HCP-1 expression in normal cells was lower than that in cancer cells, thus we concluded that the reason of cancer specific porphyrin accumulation is HCP-1 expression [12].

Nitric oxide (NO), which is a free radical gas, acts as a second messenger and has both physiological and pathological effects. The biological roles of NO and ROS are equally important [13]. Many studies have reported that NO has functions in cancer tissue related angiogenesis [14], vascular permeability [15], DNA damage [16], and apoptosis [17]. Moreover, NO regulates intracellular porphyrin accumulation in cancer cells. Indeed, we had previously reported that the fluorescence intensity of the porphyrins in the MKN45 cells increased with the administration of an NO donor after 5-aminolevulinic acid (ALA) treatment [18]. Moreover, transfection of the inducible nitric oxide synthase (iNOS) gene into the normal mouse kidney cell line HEK293T decreased the ferrochelatase activity and increased the PDT effect [19]. However, it is unclear the relationship between NO and HCP-1.

Hypoxia inducible factor (HIF)-1α is stabilized by NO and upregulates vascular endothelial growth factor (VEGF) and heme oxygenase 1, which control the vascular system [20, 21]. Both VEGF and heme oxygenase 1 stimulate angiogenesis and improve the impaired post-ischemic blood flow [22]. The HIF-1α is also related to heme biosynthesis as it upregulates the erythropoietin receptor and ferrochelatase [23]. Hence, it may also regulate HCP-1 expression to obtain heme.

In this study, we determined whether the increase in NO production upregulated the expression of HCP-1 to enhance the PDT effect by stabilizing the expression of HIF-1α.

## Materials and methods

### Materials

L-arginine, 2-methoxyestradiol (2-Me), deoxycholic acid, hydrochloric acid, and NaOH (Wako Pure Chemicals, Osaka, Japan); N6-(1-iminoethyl)-L-lysine (L-NIL) (Cayman, Michigan, USA); cell counting kit-8 (Dojindo, Tokyo, Japan); DAF-2DA (Daiichi Pure Chemicals, Tokyo, Japan); hematoporphyrin dihydrochloride (HpD), NaCl, sodium dodecyl sulfate (SDS), Trizma base, Triton X-100, and Tween 20 (Sigma-Aldrich Japan K.K., Tokyo, Japan); NuPAGE Novex 12% Bis-Tris gels (Life Technologies Japan, Tokyo, Japan); polyvinylidene difluoride (PVDF) Blocking Reagent for Can Get Signal, Can Get Signal Immunoreaction Enhancer Solution 1, and Can Get Signal Immunoreaction Enhancer Solution 2 (Toyobo, Osaka, Japan); HIF-1α, β-actin, and horseradish peroxidase (HRP)-linked anti-rabbit IgG antibodies (Cell Signaling Technology Japan, K.K., Tokyo, Japan); HCP-1 antibody (Abcam, Cambridge, U.K.); iNOS antibody (BiossAntibodies, Massachusetts, U.S.A.) and Lumina Forte western HRP substrate (EMD Millipore, Billerica, MA, USA) were purchased and used without further purification or modification.

### Cell culture

The rat cancerous gastric mucosa cell line RGK36 (S1 File), which is established in our laboratory [24], was cultured in DMEM/F12 without L-glutamine (Sigma-Aldrich Japan K.K.). The human gastric cancer cell line MKN45 was cultured in RPMI1640 (Wako Pure Chemicals). The culture media were supplemented with 10% inactivated fetal bovine serum (FBS) (Biowest LLC, Kansas City, MO) and 1% penicillin/streptomycin (Life Technologies Japan Ltd.). Cells were cultured in 5% CO_2_ cell culture incubator at 37°C.

### NO detection with DAF-2DA

The RGK36 cells were cultured in 96-well plates at 3×10^3^ cells/well and incubated overnight. Then, the cells were incubated with 10 mM L-arginine or 3 μM L-NIL, a potent and selective inhibitor of iNOS, for 24 h. The supernatant was aspirated, and the cells were incubated with 10 μM DAF-2DA for 1 h. The fluorescence intensity of DAF-2DA was measured using a Synergy H1 (BioTek Instruments Inc., Winooski, VT, USA). The excitation and emission wavelengths were 480 and 520 nm, respectively.

### Western blotting analyses

The RGK36 cells were incubated in medium containing 10 mM L-arginine, 3 μM L-NIL, or 1 μM 2-Me for 24 h. After incubation, the whole-cell lysates were prepared by rinsing the cells three times with PBS; NuPAGE LDS sample buffer was added on ice and then heated at 70°C for 10 min. For SDS-polyacrylamide gel electrophoresis, the cell lysates were added into the wells of the NuPAGE Novex 4–12% Bis-Tris gels. The gels were electrophoresed at 100 V for 70 min, and the proteins were transferred onto a PVDF membrane by electrophoresis at 1.2 mA/cm^2^ for 60 min. The membrane was blocked for 60 min with PVDF blocking reagent for the Can Get Signal kit, and probed with primary and secondary antibodies. Anti-rabbit HCP-1 (ab25134), HIF-1α (#14179), or iNOS (bs-2072R) antibodies (1:1000) were added to Can Get Signal Immunoreaction Enhancer Solution 1. The membrane was exposed to the solution overnight. After the primary antibody solution was aspirated, the membrane was washed three times with 1x Tris-buffered saline containing Tween 20. The secondary HRP-linked anti-rabbit IgG antibody (#7074) was added to the Can Get Signal Immunoreaction Enhancer Solution 2. The solution was exposed to the membrane for 60 min. Chemiluminescence was used to develop the membrane. Images of the blots were captured by ImageQuant LAS4000 (GE Health Care Japan, Tokyo, Japan). β-Actin (#4967) was used as the control for protein loading.

### Cellular uptake of HpD

The RGK36 and MKN45 cells were incubated overnight in 12-well plates at 5×10^4^ cells/well, each. Cells were then incubated with 10 mM L-arginine, 3 μM L-NIL, or 1 μM 2-Me for 24 h. After incubation, 20 μM HpD was added to the cells and incubated for 6 h. The cells were rinsed with PBS and lysed in 100 μL RIPA buffer containing 25 mM Tris-HCl (pH 7.6), 150 mM NaCl, 1% (v/v) Triton X-100, 0.1% (w/v) SDS, and 0.2% (w/v) deoxycholic acid. Cell homogenates were transferred to a 96-well plate. The fluorescence intensity of HpD was measured using a Varioskan microplate reader. The excitation and emission wavelengths were 415 and 625 nm, respectively.

### Cell viability assay after PDT

The RGK36 and MKN45 cells were incubated overnight in 96-well plates at 1×10^3^ cells/well, each, and then incubated with 10 mM L-arginine, 3 μM L-NIL, or 1 μM 2-Me for 24 h. After the treatments, the cells were incubated with 20 μM HpD for 24 h and rinsed with PBS. Fresh medium without phenol red was added. Cells were irradiated by excimer dye laser light (630 nm, 0.5 J/cm^2^) for PDT by using PDT EDL-1 (Hamamatsu Photonics K.K., Hamamatsu, Japan) and incubated for 24 h. The medium was replaced with fresh medium containing 10% Cell Counting Kit-8, and further incubated. The absorbance at 450 nm was measured on a DTX880 multi-mode microplate reader.

### Statistical analysis

Data are expressed as the mean ± SD and were assessed by analysis of variance (ANOVA). Individual groups were compared by Student’s t-test or Tukey’s test, with p<0.05 considered statistically significant.

## Results

### Intracellular NO concentration was regulated by L-arginine and/ or L-NIL

**Fig 1:**
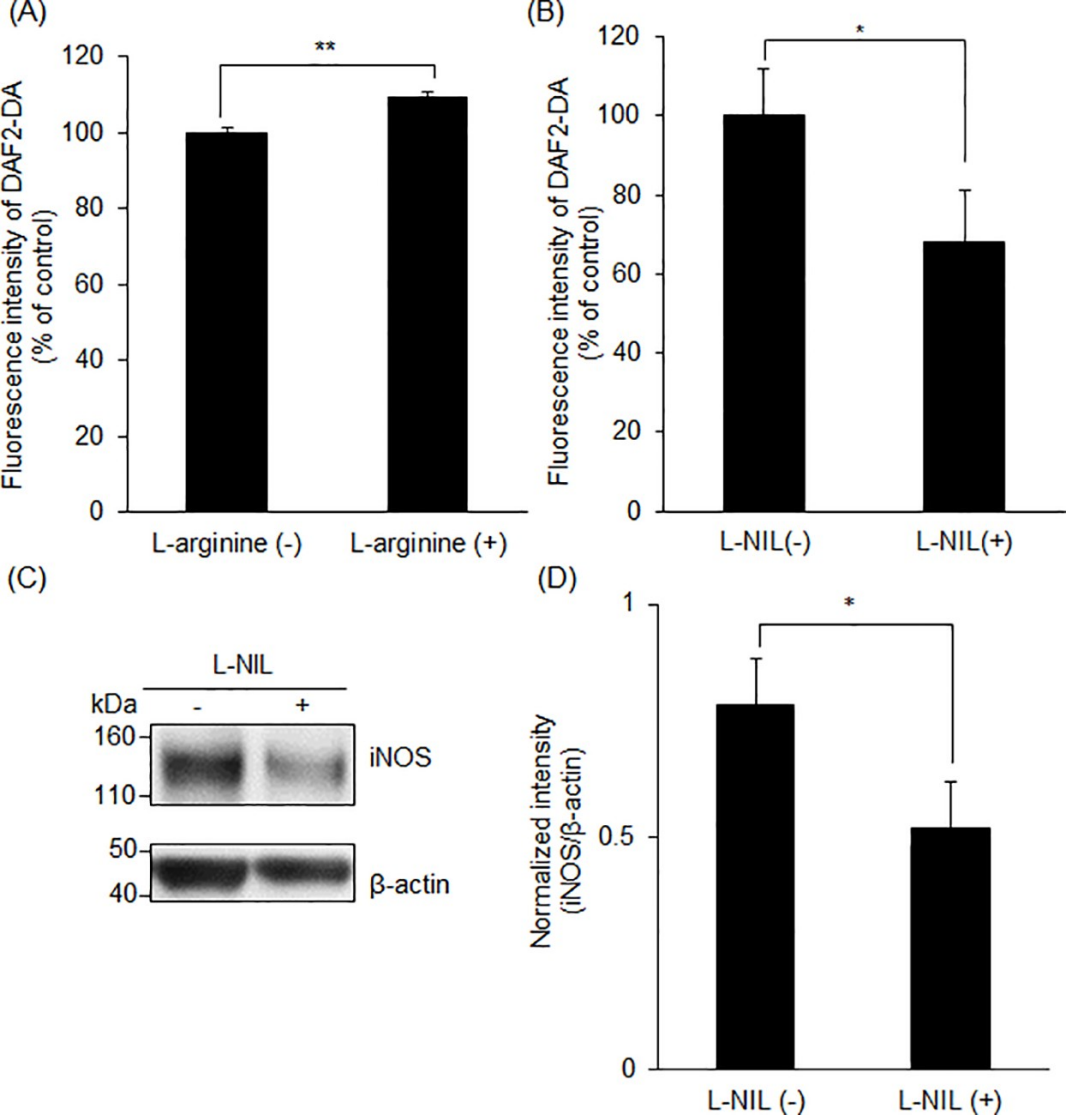
NO concentration and iNOS expression in RGK36 cells. Intracellular NO concentration increased with L-arginine (A) while decreased with L-NIL (B). Data are expressed as the mean ± SD (n = 4). *p < 0.05, **p < 0.01. iNOS expression decreased with L-NIL (C, D). Data are expressed as the mean ± SD (n = 3). *p < 0.05.

The intracellular NO concentration was determined using DAF-2DA. L-arginine was used as a precursor for NO production, whereas L-NIL was used as iNOS inhibitor. The fluorescence intensity of the dye significantly increased in 10 mM L-arginine-treated cells, while it significantly decreased in 3 μM L-NIL-treated cells compared to control cells (Fig 1A and 1B). Moreover, L-NIL treatment induced the downregulation of iNOS expression (Fig 1C and 1D). These results indicate that the intracellular NO concentration was increased by L-arginine and decreased by L-NIL.

### HIF-1α and HCP-1 protein expression increased by L-arginine treatment and decreased by L-NIL or 2-Me

**Fig 2:**
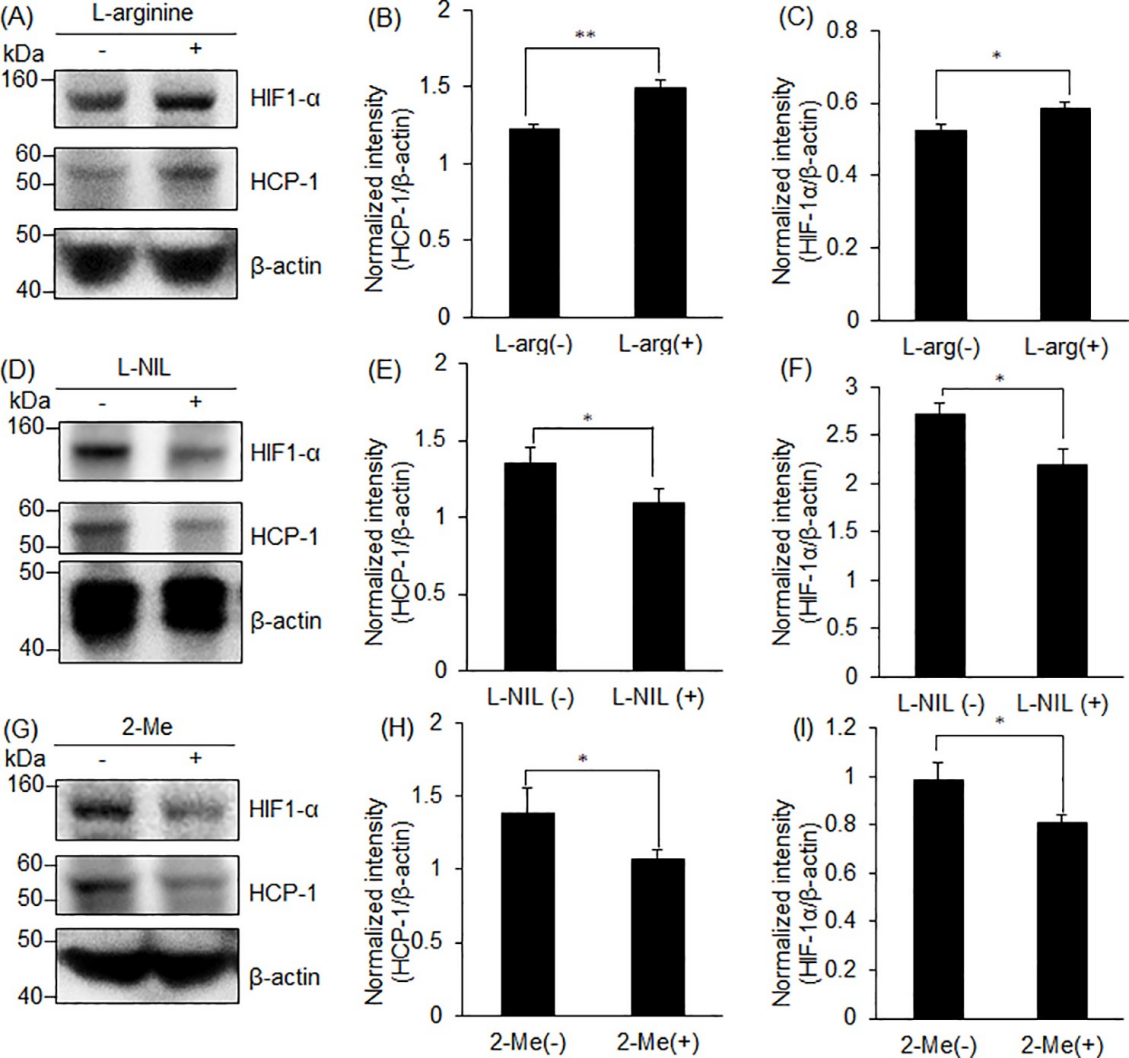
Expression of HCP-1 and HIF-1α in RGK36 cells. HCP-1, HIF-1α, and β-actin were detected by western blot. Both HCP-1 and HIF-1α expression was increased with L-arginine (A-C), while it decreased with L-NIL (D-F) or 2-Me (G-I). Data are expressed as the mean ± SD (n = 3). *p < 0.05, **p < 0.01.

The RGK36 cells were treated with L-arginine, L-NIL, or 1 μM 2-Me for 24 h and then analyzed for the expression of HIF-1α and HCP-1 by western blotting. Both HIF-1α and HCP-1 expression in cells treated with L-arginine was significantly higher than that in cells treated without L-arginine (Fig 2A–2C). The expression of both the proteins in L-NIL- treated cells, where the production of NO was inhibited, was significantly decreased (Fig 2D–2F). 2-Me is known as a HIF-1α inhibitor. The HIF-1α expression in 2-Me-treated cells was significantly decreased (Fig 2G and 2H). Moreover, the HCP-1 expression also decreased by 2-Me treatment (Fig 2G and 2I).

### The fluorescence intensity of HpD increased in L-arginine treatment cells whereas decreased in L-NIL or 2-Me

**Fig 3:**
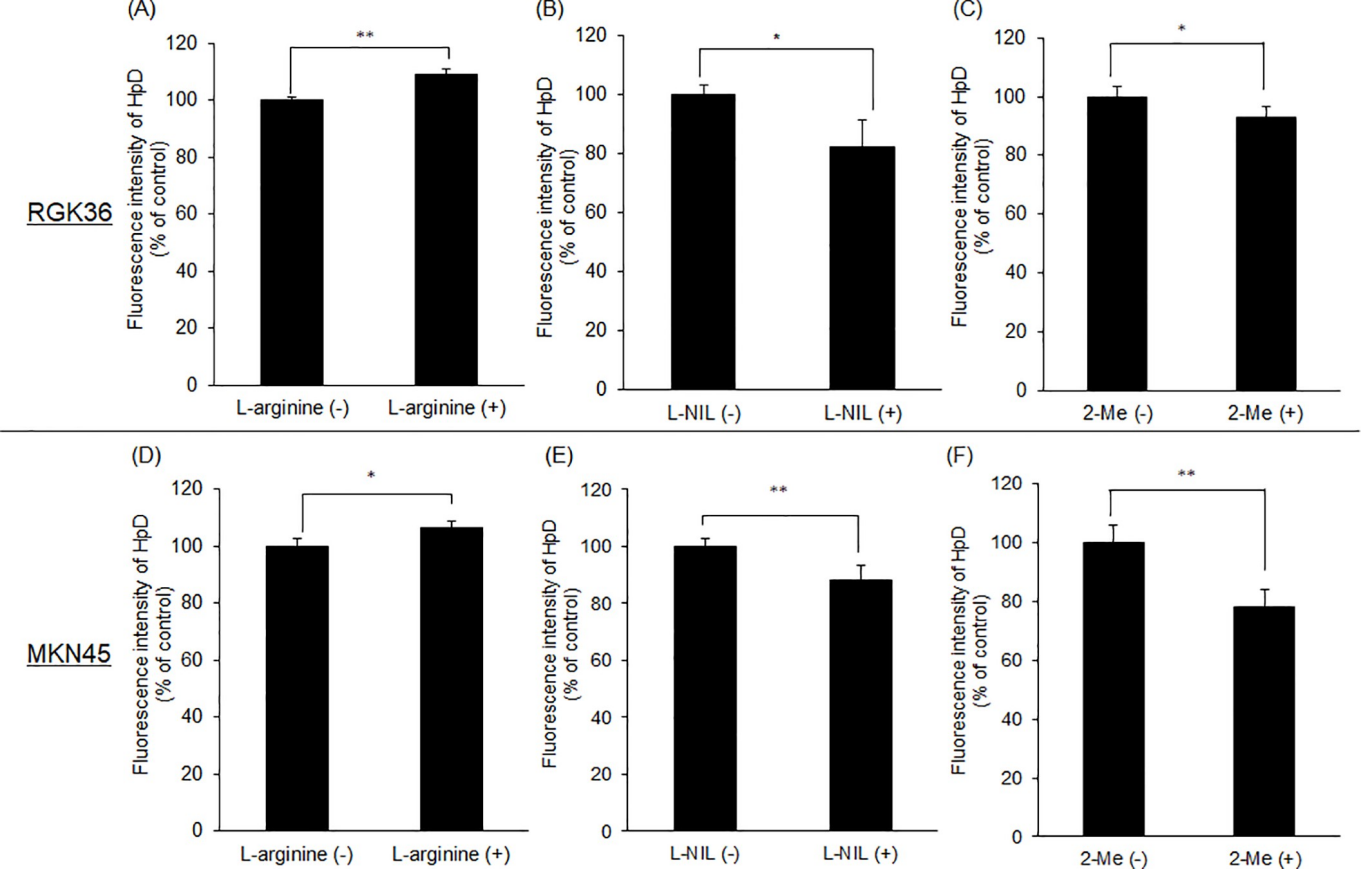
Intracellular HpD accumulation in RGK36 and MKN45 cells. Cells were treated with 10 mM L-arginine (A, D), 3 μM L-NIL (B, E), or 1 μM 2-Me (C, F) for 24 h. Then, the cells were exposed to culture medium containing 20 μM HpD for 6 h. Data are expressed as the mean ± SD (n = 4). *p < 0.05, **p < 0.01.

The RGK36 cells and the MKN45 cells were treated with L-arginine, L-NIL, or 2-Me for 24 h, and then the cells were treated with 20 μM HpD for 6 h. The intracellular fluorescence intensity of HpD was measured with a microplate reader. In both RGK36 and MKN45 cells, the intracellular fluorescence intensity of HpD was higher after the L-arginine treatment, while treatment with L-NIL and 2-Me significantly decreased the intensity compared to the control cells (Fig 3A–3F).

### Cytotoxicity of PDT was enhanced by L-arginine while suppressed by L-NIL or 2-Me

**Fig 4:**
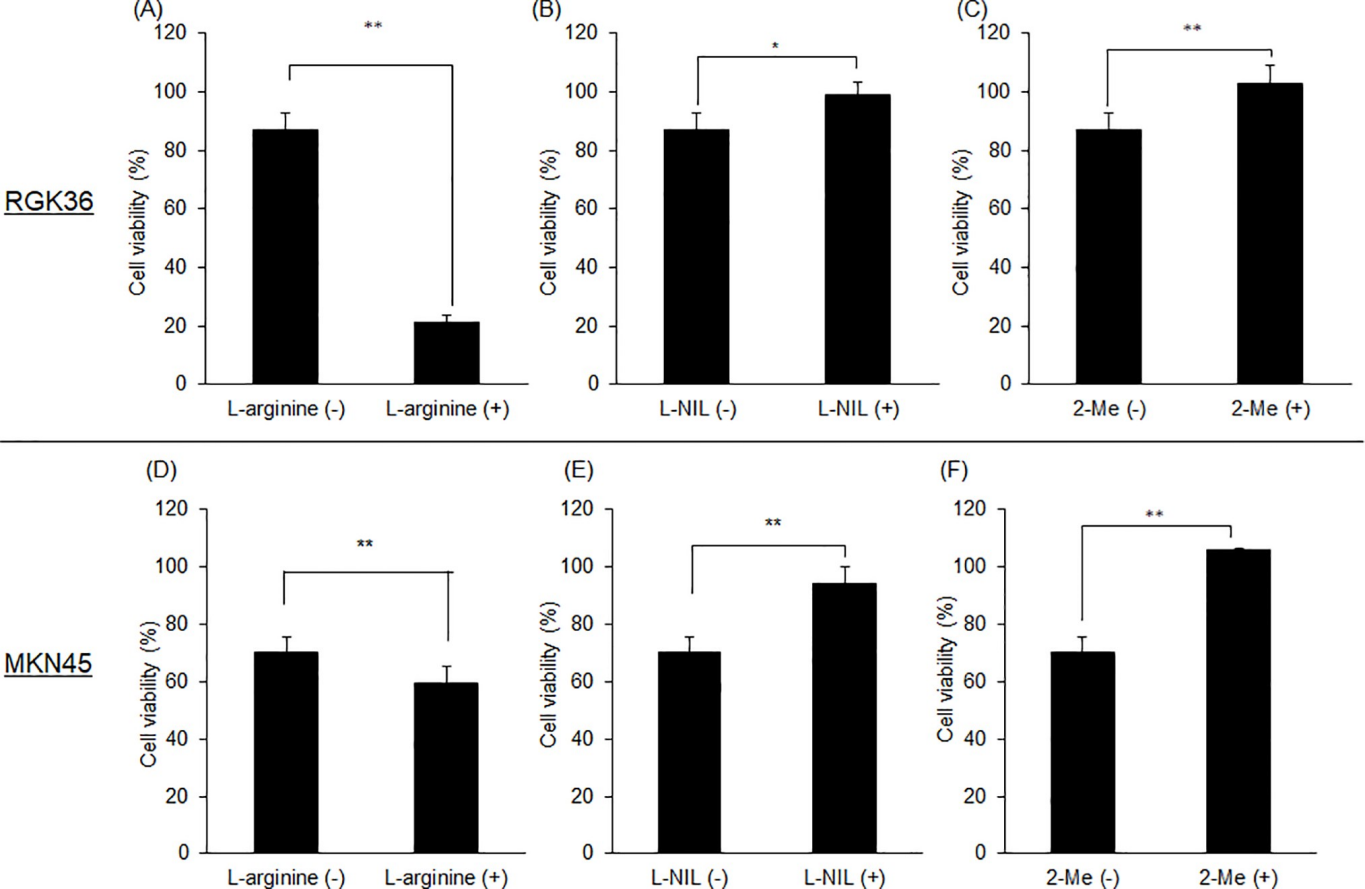
The PDT effect was examined after exposure to HpD in RGK36 and MKN45 cells. Cells were treated with 10 mM L-arginine (A, D), 3 μM L-NIL (B, E), or 1 μM 2-Me (C, F) for 24 h. Cell viability was evaluated after HpD exposure and laser irradiation. Data are expressed as the mean ± SD (n = 4). *p < 0.05, **p < 0.01.

The RGK36 cells and the MKN45 cells were treated with L-arginine, L-NIL, or 2-Me for 24 h; then the cells were treated with 20 μM HpD for 24 h. The cells were irradiated with 630 nm laser. Cellular injuries after the PDT effect was evaluated with the WST assay. In case of RGK36, the cytotoxicity of PDT was accelerated in the L-arginine-treated cells, compared to the non-treated cells (Fig 4A). However, the PDT effect for cells treated with L-NIL or 2-Me was suppressed (Fig 4B and 4C). The same phenomenon was also induced in MKN 45 for the effect of L-arginine, L-NIL or 2-Me treatments.

### Porphyrin accumulation was inhibited by 2-Me even though the cells were treated with L-arginine

**Fig 5:**
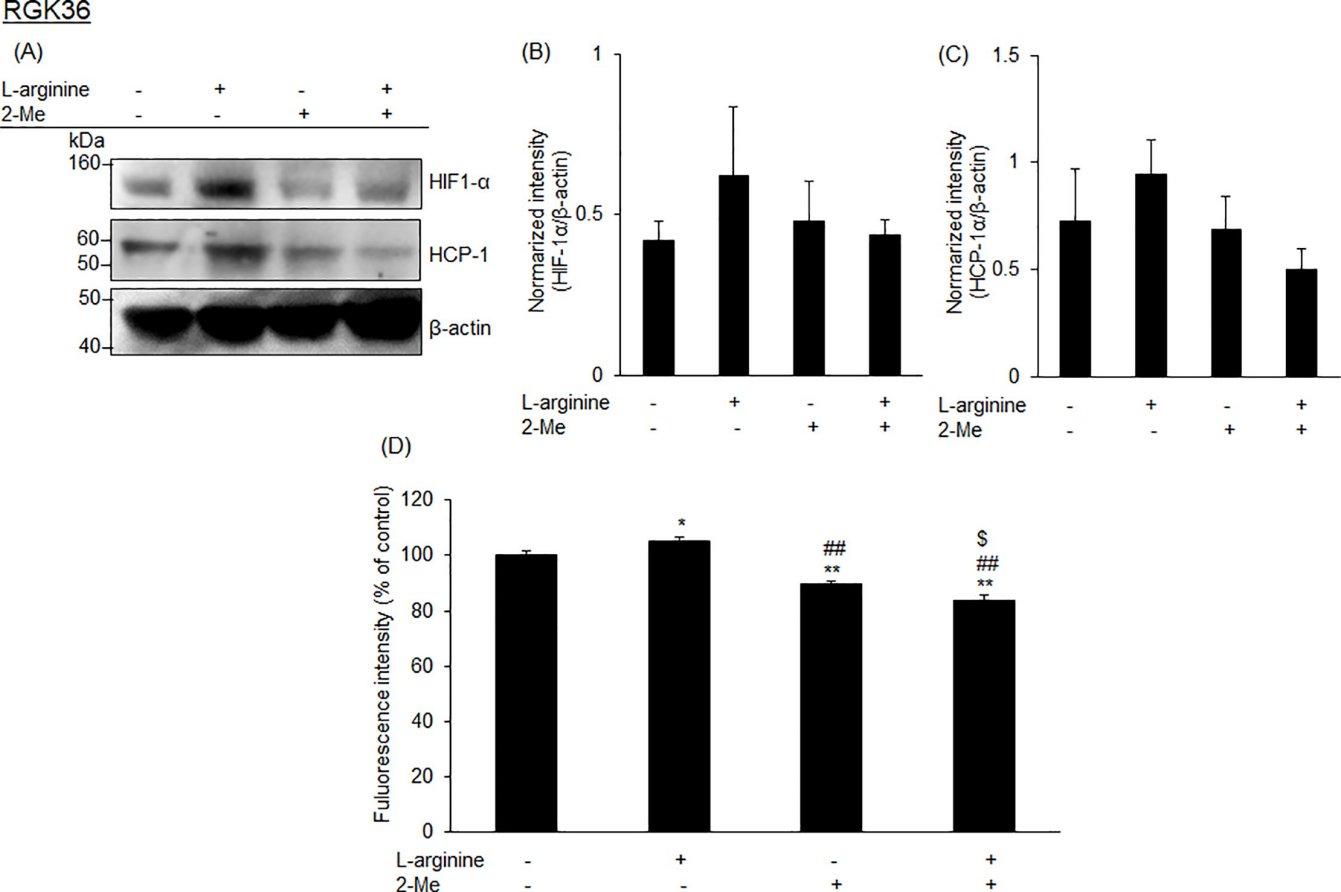
The activity of L-arginine was inhibited by 2-Me. Expression of HCP-1 and HIF-1α in RGK36 cells was inhibited by 1 μM 2-Me (A-B). Data are expressed as the mean ± SD (n = 3). Intracellular HpD accumulation was also suppressed by 1 μM 2-Me (D). Data are expressed as the mean ± SD (n = 4). *p < 0.05 and **p < 0.01 vs control. ##p < 0.01 vs L-arginine. $p < 0.05 vs 2-Me.

Cells were treated with L-arginine, 2-Me, or both for 24 h and then analyzed for HIF-1α and HCP-1 expression by western blotting. L-arginine treatment increased the HCP-1 expression. However, the combination of L-arginine and 2-Me could not increase the expression. Similarly, L-arginine treatment increased HIF-1α expression, while the combination treatment could not increase (Fig 5A–5C). The intracellular fluorescence intensity of HpD in L-arginine-treated cells became higher in comparison to the control cells. However, the fluorescence intensity in the combination treated-cells became lower in comparison to the control cells (Fig 5D).

## Discussion

In this study, we demonstrated for the first time that NO increased the intracellular porphyrin accumulation by upregulation of HCP-1 expression via HIF-1α stabilization. It is reported that HCP-1 transports not only heme but also porphyrins [11, 12]. NO regulates the blood flow to obtain O_2_; thus, we hypothesized that NO also regulates HCP-1 expression to increase the intracellular heme concentration. NO is produced through the conversion of L-arginine to L-citrulline by the enzyme nitric oxide synthase (NOS) [25]. In this study, we used L-arginine to enhance the NO production, whereas L-NIL was used to inhibit the NO production. At a concentration of 0.4 to 3.3 μM, L-NIL works as a selective iNOS inhibitor [26]. Therefore, the intracellular NO concentration increased by L-arginine and decreased by L-NIL (Fig 1A–1D). We concluded that HCP-1 expression could be regulated by L-arginine or L-NIL. L-arginine, the NO producer, upregulated HCP-1 expression, while L-NIL, the NO inhibitor, downregulated HCP-1 expression (Fig 2A, 2B, 2D and 2E). Taken together, we concluded that regulation of intracellular NO influenced HCP-1 expression.

We also investigated the relationship between NO and HIF-1α. In normoxia, HIF-1α is hydroxylated by prolyl hydroxylase (PHD) and binds to the von Hippel Lindau protein. Under this condition, HIF-1α is ubiquitinated and degraded by the 26S proteasome [27]. Asparagine-803 of HIF-1α is also hydroxylated by an oxygen-dependent asparagine hydroxylase, referred to as factor-inhibiting HIF-1 (FIH). The recruitment of CBP/p300 is prevented by the hydroxylated asparagine residue, thereby inhibiting transactivation by the stabilized HIF-1α. In hypoxia, HIF-1α is stabilized as a result of inhibition of prolyl hydroxylase. Even in normoxia, HIF-1α can be stabilized by NO, which inhibits the PHD activity through the nitrosylation of cysteine residues [28]. The FIH also appears to be inhibited by NO, and the mechanism is likely similar to that of PHD. HIF-1α expression was increased by L-arginine and decreased by L-NIL, similarly to HCP-1 expression (Fig 2A, 2C, 2D and 2F). Regulation of NO concentration by L-arginine or L-NIL affected the expression of HIF-1α, which when stable can accelerate HCP-1 expression. 2-Me is a derivative of estradiol and downregulates HIF-1α at the post-transcriptional level [29, 30]. After the 2-Me treatment, both HIF-1α and HCP-1 expression decreased in RGK36 cells (Fig 2G–2I). These results also indicate that HIF-1α regulates HCP-1 expression.

We investigated the relationship between porphyrin accumulation and the PDT effect. The HCP-1 expression was increased in the L-arginine-treated cells, while L-NIL or 2-Me treatment decreased the expression. We proposed that the intracellular porphyrin accumulation can be controlled with L-arginine, L-NIL, and 2-Me. Fluorescence intensity of porphyrins was significantly higher in L-arginine-treated cells than control cells (Fig 3A). However, the fluorescence intensity decreased in L-NIL or 2-Me-treated cells (Fig 3B and 3C). In other words, intracellular porphyrin accumulation increased by L-arginine and decreased by L-NIL or 2-Me. Moreover, cytotoxicity of PDT increased in L-arginine treatment while it decreased in L-NIL or 2-Me (Fig 4A–4C). Since inhibition of HIF-1α with 2-Me decreased porphyrin accumulation and PDT effect, we proposed that HCP-1 expression was regulated by NO via HIF-1α stabilization. HCP-1 expression, upon treatment with the combination of L-arginine and 2-Me, was lower than that upon treatment with L-arginine alone. The HIF-1α expression was decreased by L-arginine and 2-Me, similar to the HCP-1 expression. However, there was no significant difference between each group (Fig 5A–5C). On the other hand, intracellular porphyrin accumulation was inhibited by L-arginine and 2-Me (Fig 5D). Based on these results, we considered that the HCP-1 expression was regulated by NO via HIF-1α stabilization and assumed that the strong PDT effect by L- arginine may attenuate in combination with 2-Me.

We previously reported that iNOS overexpression decreased the ferrochelatase activity of HEK293T cells [19]. Moreover, PDT-induced cell death in iNOS-expressing HEK293T cells was greater than that in mock transfected cells. Since NO participates in porphyrin accumulation via ferrochelatase inactivation, we assumed that NO would be involved in the HCP-1 expression. In this study, we showed that HCP-1 expression increased after treatment of RGK36 cells with L-arginine, whereas it decreased after treatment with L-NIL or 2-Me. HIF-1α upregulate the ferrochelatase gene expression [31]. However 2-Me treatment decreased intracellular porphyrin accumulation from in this study. We showed the level and activity of ferrochelatase were decreased by the expression of iNOS, however the degree of the decrease of the enzyme activity was somewhat different from that of the enzyme level [18]. Thus we regarded that upregulation of ferrochelates does not necessarily directly increase porphyrin accumulation. These results suggest that the accumulation of HpD in response to NO involved not only ferrochelatase inhibition but also HCP-1 over-expression. This phenomenon also accelerated the PDT effect.

In this study, we used both rat and human gastric cancer cells, RGK36 and MKN45. In MKN45 cells, the fluorescence intensity of porphyrin increased by L-arginine and decreased by L-NIL or 2-Me. Thus, the intracellular porphyrin accumulation was regulated by these treatments (Fig 3D–3F). The cytotoxicity of PDT was also enhanced by L-arginine and decreased by L-NIL or 2-Me in the RGK36 cells (Fig 4D–4F). Thereby PDT cytotoxicity can be enhanced by increasing the NO production in clinical cases.

**Fig 6:**
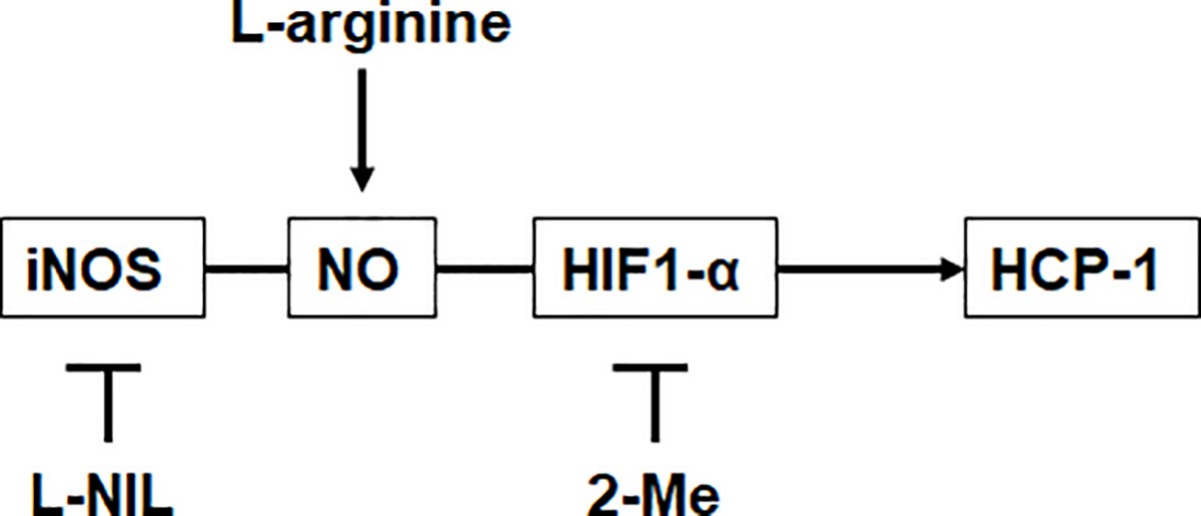
The mechanism of regulation of HCP-1 expression. HCP-1 expression increase by L-arginine, while it decrease by L-NIL or 2-Me.

In conclusion, NO stabilizes the HIF-1α to accelerate HCP-1 expression, resulting in enhanced intracellular porphyrin accumulation that improves the PDT effect (Fig 6).

## Supporting information

S1 File Cell lines. (DOCX)
