# Supplementary material for: Nitric oxide regulates the expression of heme carrier protein-1 via hypoxia inducible factor-1α stabilization
Source: PLoS One. 2019 Sep 12;14(9):e0222074. doi: 10.1371/journal.pone.0222074 (PMC6742216; doi:10.1371/journal.pone.0222074)
Supplement: S1 File — (DOCX) [file pone.0222074.s001.docx]

**Cell lines**

In this study, we used the rat cancerous gastric mucosa cell line RGK36, which is established in our laboratory (Shimokawa O, Matsui H, Nagano Y, Kaneko T, Shibahara T, Nakahara A, et al. Neoplastic transformation and induction of H+,K+ -adenosine triphosphatase by N-methyl-N'-nitro-N-nitrosoguanidine in the gastric epithelial RGM-1 cell line. In vitro cellular & developmental biology Animal. 2008; 44: 26-30.) First, we established RGK1 from rat gastric mucosa cell line RGM1 using 1-Methyl-3-nitro-1-nitrosoguanidine. We established RGK36 by limiting dilution culture of RGK1.
